# Glucose restriction induces transient G2 cell cycle arrest extending cellular chronological lifespan

**DOI:** 10.1038/srep19629

**Published:** 2016-01-25

**Authors:** Fumie Masuda, Mahiro Ishii, Ayaka Mori, Lisa Uehara, Mitsuhiro Yanagida, Kojiro Takeda, Shigeaki Saitoh

**Affiliations:** 1 Institute of Life Science, Kurume University, Hyakunen-Khoen 1-1, Kurume, Fukuoka 839-0864, Japan; 2 Department of Biology, Faculty of Science and Engineering, Konan University, 8-9-1 Okamoto, Higashinada-ku, Kobe 658-8501, Japan; 3 Okinawa Institute of Science and Technology Graduate School, Tancha 1919-1, Onna, Okinawa 904-0495, Japan; 4 Institute for Integrative Neurobiology, Konan University, 8-9-1 Okamoto, Higashinada-ku, Kobe 658-8501, Japan

## Abstract

While glucose is the fundamental source of energy in most eukaryotes, it is not always abundantly available in natural environments, including within the human body. Eukaryotic cells are therefore thought to possess adaptive mechanisms to survive glucose-limited conditions, which remain unclear. Here, we report a novel mechanism regulating cell cycle progression in response to abrupt changes in extracellular glucose concentration. Upon reduction of glucose in the medium, wild-type fission yeast cells undergo transient arrest specifically at G2 phase. This cell cycle arrest is dependent on the Wee1 tyrosine kinase inhibiting the key cell cycle regulator, CDK1/Cdc2. Mutant cells lacking Wee1 are not arrested at G2 upon glucose limitation and lose viability faster than the wild-type cells under glucose-depleted quiescent conditions, suggesting that this cell cycle arrest is required for extension of chronological lifespan. Our findings indicate the presence of a novel cell cycle checkpoint monitoring glucose availability, which may be a good molecular target for cancer therapy.

Cell growth, division and differentiation in eukaryotes are regulated by various compounds in the microenvironment surrounding the cells, such as growth factors and nutrients. In unicellular eukaryotes, nutrients in the medium are major determinants of the timing of cell division (i.e., cell cycle progression) and differentiation. In the fission yeast, *Schizosaccharomyces pombe*, for example, the depletion of nitrogen sources accelerates cell cycle progression temporarily, and then arrests the cells in G1 phase after two rounds of cell division^1^,^2^,^3^. *S. pombe* cells subsequently undergo sexual differentiation in the presence of mating pheromones, whereas the cells enter the quiescent (G0) state in their absence, which confers resistance to various types of stress^4^,^5^. In multicellular organisms, such as fruit flies, the availability of nutrients during larval development determines the size of the body by modulating the sizes and the numbers of cells via nutrient-sensing signalling cascades involving the target of rapamycin (TOR) kinase and insulin-like growth factors^6^,^7^,^8^. Thus, in both unicellular and multicellular eukaryotes, the rate and the timing of cell cycle progression are regulated in response to changes in extracellular nutritional status.

The TOR kinases, which form two distinct complexes, TORC1 and TORC2, are suggested to play a pivotal role in cellular response to extracellular nutrients, such as amino acids^9^,^10^,^11^,^12^,^13^. In *S. pombe*, the TOR complexes control cell growth, division and sexual differentiation in response to limitation of nitrogen sources and/or glucose^14^,^15^,^16^,^17^,^18^,^19^,^20^,^21^,^22^,^23^,^24^,^25^. Very recently, we demonstrated that *S. pombe* TORC2, but not TORC1, is required for proper localization of the high-affinity glucose transporter, Ght5, transcription of which is elevated upon glucose restriction in a manner dependent on calcium/calmodulin dependent kinase kinase (CaMKK)^26^,^27^. In *S. pombe*, TORC2 and CaMKK are required for enhancement of glucose uptake, and thus for vigorous cell proliferation, under glucose-limited conditions. Intriguingly, when *S. pombe* cells are transferred from high-glucose (111 mM) medium to low-glucose (4.4 mM) medium, they stop dividing transiently before resuming rapid proliferation^26^,^28^. These findings indicate that reduction of extracellular glucose triggers large-scale remodelling in the molecular machinery involved in regulation of glucose transport and metabolism, and cell proliferation.

Periodic activation and inactivation of cyclin-dependent protein kinases (CDKs) drive the progression of the cell cycle in eukaryotes. While higher eukaryotes possess multiple types of CDK, each of which is responsible for transitions of different stages of the cell cycle, the single CDK (Cdc2/CDK1), which is thought to be the prototype of the CDKs, controls the entire cell cycle in *S. pombe*, in association with different stage-specific cyclins^29^,^30^. Studies using various model organisms, including fission yeast, have revealed the core mechanism underlying the regulation of cell cycle progression; the activities of CDKs are regulated by association with the cyclin subunits and phosphorylation. The evolutionarily conserved tyrosine kinase(s) and the counteracting phosphatase, Wee1 (and a related kinase, Mik1) and Cdc25, which were originally identified in fission yeast, control the activity of Cdc2/CDK1 through the inhibitory phosphorylation of its tyrosine 15 (Tyr 15) residue^31^,^32^,^33^,^34^,^35^,^36^. Tyr 15 phosphorylation by Wee1 inactivates Cdc2 and prevents the G2/M transition, while its dephosphorylation by Cdc25 promotes entry into M phase. Thus, the balance between Wee1 and Cdc25 determines the timing of the onset of M phase.

Cell cycle progression is regulated in response to various types of stress. Genotoxic stresses, such as DNA damage and incomplete replication, activate the checkpoint, which prevents cell cycle progression until the stresses are removed^37^,^38^,^39^. In response to these genotoxic stresses, the evolutionarily conserved DNA structure checkpoint signalling cascade, upstream of which ATM (Ataxia telangiectasia mutated) and ATR (ATM and Rad3-related) kinases act, ultimately inhibit Cdc25, so that the cells are arrested in G2 phase. Defects in the checkpoints result in the rapid loss of genomic integrity and cell viability in the presence of genotoxic stresses. In contrast, the molecular mechanism underlying the modulation of cell cycle progression in response to nutritional stresses, such as glucose restriction, is largely unknown, and the physiological significance of such a mechanism, if it exists, remains to be elucidated.

Here, we explore the mechanism underlying the regulation of cell cycle progression under low glucose conditions, and show that glucose restriction causes transient G2 arrest in a manner dependent on Wee1 kinase. Mutant cells lacking Wee1 failed to cease cell cycle progression at G2 phase upon glucose restriction, and lost cell viability after glucose depletion. Our findings indicate that Wee1-dependent G2 arrest is a novel checkpoint control in response to glucose restriction, which extends the chronological lifespan under conditions of glucose depletion.

## Results

### Glucose restriction causes transient cell cycle arrest in G2 phase

**Figure 1:**
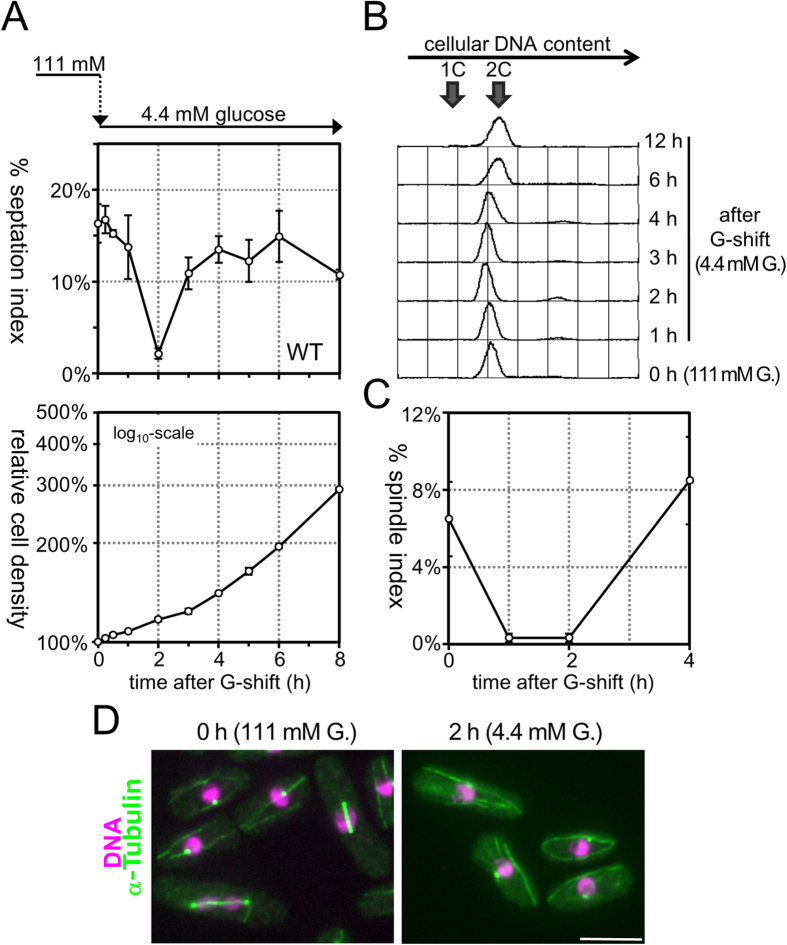
Glucose restriction causes transient cell cycle arrest in G2 phase. (**A**) Time courses of septation index (upper panel) and relative cell density (lower panel) of WT fission yeast cells that were transferred from high-glucose (111 mM) to low-glucose (4.4 mM) medium at time = 0 were examined. The averages of three independent experiments are shown, and the bars represent standard deviation (SD). (**B**) The distributions of cellular DNA content were measured in WT cells by flow cytometry analysis after transfer from high-glucose to low-glucose medium at time = 0. The positions of the peak of 1C (before replication) and 2C (after replication) DNA cells are indicated by arrows. (**C**) The time course of changes in the proportion of cells with a mitotic spindle (spindle index) was examined in WT cells after transfer to low-glucose medium. The experiments were repeated independently three times, and the averages and SD are shown. (**D**) Fluorescence microscopic images of WT cells expressing GFP-fused α-tubulin and Sid4 before (left panel) and 2 hours after transfer to low-glucose medium (right panel) are shown. GFP fluorescence is pseudocoloured green, whereas the nuclear DNA stained by DAPI is shown in magenta. Bars, 5 μm.

While regular laboratory media for *S. pombe* contain 2–3% (111–167 mM) glucose, the wild-type (WT) cells proliferate in medium containing only 0.08% (4.4 mM) glucose, which is equivalent to that in normal human blood, at a division rate similar to that in regular high-glucose medium. When transferred from high-glucose (2%, 111 mM) to low-glucose (0.08%, 4.4 mM) medium, cells stop dividing for a period of 1–2 generations (3–5 hours at 26 °C), and then resume vigorous cell division in a manner dependent on full mitochondrial function^26^,^27^,^28^,^40^. To gain mechanistic insight into cell division control in response to limitation of extracellular glucose, we monitored cell cycle progression in WT cells transferred from high-glucose to low-glucose medium by measuring the proportion of cells with a septum (septation index, %SI), which is a useful hallmark of cytokinesis (Fig. 1A). While %SI was maintained at ~15% in an asynchronous population of cells growing in synthetic Edinburgh minimal medium 2 (EMM2 medium) containing a high glucose concentration (111 mM) at 26 °C, it dropped to 2.2% at 2 hours after transfer to low-glucose (4.4 mM) EMM2 medium. The %SI then returned to a level comparable to that in high-glucose medium, as the cell number resumed increasing at a rate of 3.8 hours per division, which was virtually identical to the rate in high-glucose medium^28^. This observation indicated that acute restriction of extracellular glucose caused transient cell cycle arrest before the onset of cytokinesis. Notably, the length of cells did not increase after the shift to low-glucose medium, but rather became shorter, suggesting that cell growth (i.e., the extension of cell length) was inhibited during this arrest caused by glucose restriction, unlike cell cycle arrest due to stresses causing DNA damage and/or incomplete DNA replication, even in the presence of which the WT cells continued to grow^41^,^42^.

To determine in which phase of the cell cycle the cells were transiently arrested upon glucose restriction, we performed flow cytometry analysis to measure cellular DNA content (Fig. 1B). The histograms showed the distribution of the DNA content per cell before/after transfer from high-glucose to low-glucose medium. A single peak appeared before transfer (time = 0 hour), as most WT *S. pombe* cells growing asynchronously in regular high-glucose EMM2 medium are in G2^43^,^44^. After transfer to low-glucose medium (time = 1–6 hours), only one peak at the 2C DNA content was still present; even at the time point when %SI became minimal (time = 2 hours), no other sub-peaks appeared, indicating that transient cell cycle arrest due to glucose restriction occurred after the completion of DNA replication, i.e., in G2 or M phase. We then measured the proportion of cells with mitotic spindle (spindle index, %SpI) after transfer to low-glucose medium using a WT strain expressing α-tubulin and Sid4 labelled with green fluorescent protein (GFP), which allowed visualization of the spindle microtubules and poles, respectively (Fig. 1 C,D). The %SpI, which was 6.5% before transfer, dropped to nearly 0% transiently at 1–2 hours after transfer to low-glucose medium and then recovered to the same level as before the shift at 4 hours (Fig. 1C). The absence of cells with a mitotic spindle at 2 hours indicated that the cells were arrested before the onset of M phase. Taken together, these observations suggested that glucose restriction caused temporary arrest specifically at G2 phase. It should be noted that this G2 arrest was not caused by hypotonic shock due to glucose restriction; %SI still dropped after transfer to low-glucose medium supplemented with 107 mM sorbitol, which compensated the reduction of osmotic pressure (Supplementary Fig. 1).

### Wee1 is required for transient G2 arrest due to glucose restriction

**Figure 2:**
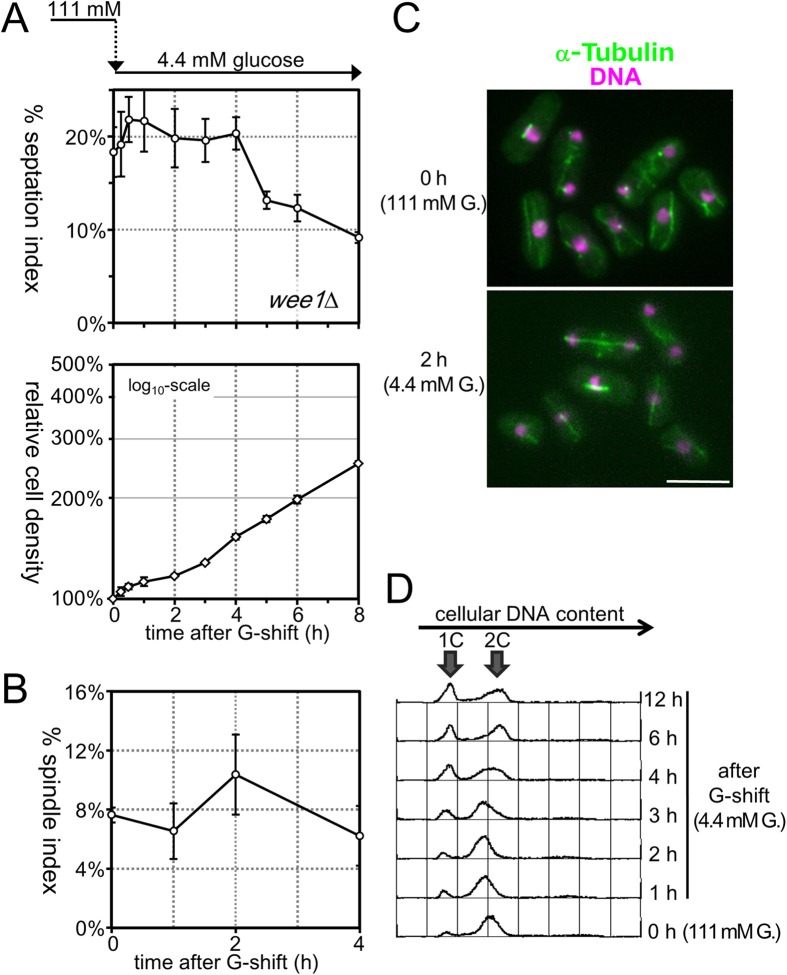
Mutant cells lacking *wee1*^+^ failed to be arrested at G2 phase after glucose restriction. (**A**) Time courses of changes in septation index (upper panel) and relative cell density (lower panel) of mutant cells lacking the *wee1*^+^ gene (*wee1*Δ) that were transferred from high-glucose (111 mM) to low-glucose (4.4 mM) medium at time = 0 were examined. The averages of three independent experiments are shown, and the bars indicate ±SD. (**B**) The time course of changes in the spindle index was examined in *wee1*Δ cells after transfer to low-glucose medium. The experiments were repeated independently three times, and the averages ±SD are shown. (**C**) Fluorescence microscopic images of *wee1*Δ cells expressing GFP-fused α-tubulin before (upper panel) and 2 hours after transfer to low-glucose medium (lower panel) are shown. GFP fluorescence is pseudocoloured green, whereas the nuclear DNA stained by DAPI is shown in magenta. Bars, 5 μm. (**D**) The distributions of cellular DNA content were measured in *wee1*Δ cells by flow cytometry analysis after transfer from high-glucose to low-glucose medium at time = 0. The positions of the peak of 1C (before replication) and 2C (after replication) DNA cells are indicated by arrows.

To identify genes required for cell proliferation specifically under glucose-limited conditions, we systematically screened a *S. pombe* gene deletion strain library for mutant strains defective in cell proliferation on low-glucose medium. Approximately 150 gene deletion mutant strains were identified, including six mutants lacking a cycle-related gene (*mcl1*^+^, *mpr1*^+^, *rad1*^+^, *rad26*^+^, *sum2*^+^ or *wee1*^+^)^27^. To examine whether these six genes were involved in transient G2 arrest upon glucose restriction, %SI was measured in mutant cells lacking each one of these cell cycle-related genes after transfer to low-glucose medium. Among these genes, the *wee1*^+^ gene was suggested to be essential for cell cycle control in response to glucose restriction; in mutant cells lacking *wee1*^+^ (*wee1*Δ), %SI remained high after transfer to low-glucose medium, although the cell number stopped increasing transiently (Fig. 2A). This result suggested that, when the extracellular glucose concentration was reduced abruptly, the *wee1*Δ cells ceased dividing at any cell cycle stage presumably due to energy (i.e., ATP) shortage. Consistent with this suggestion, %SpI representing the proportion of mitotic cells remained high in the *wee1*Δ mutant even after transfer to low-glucose medium, and cells in early- to late-mitosis were frequently observed in *wee1*Δ (Fig. 2B,C), in marked contrast to WT controls in which mitotic cells were seldom observed at 1–2 hours after transfer to low-glucose medium (Fig. 1C,D). Measurement of cellular DNA content revealed that 1C DNA cells became prominent in *wee1*Δ after prolonged incubation in low-glucose medium (Fig. 2D), indicating that cells before the onset of DNA replication, completion of which requires a large amount of ATP, were gradually accumulated in this mutant strain during cultivation under glucose-limited conditions. Taken together, these observations indicated that Wee1 is essential for G2 cell cycle arrest in response to glucose restriction.

**Figure 3:**
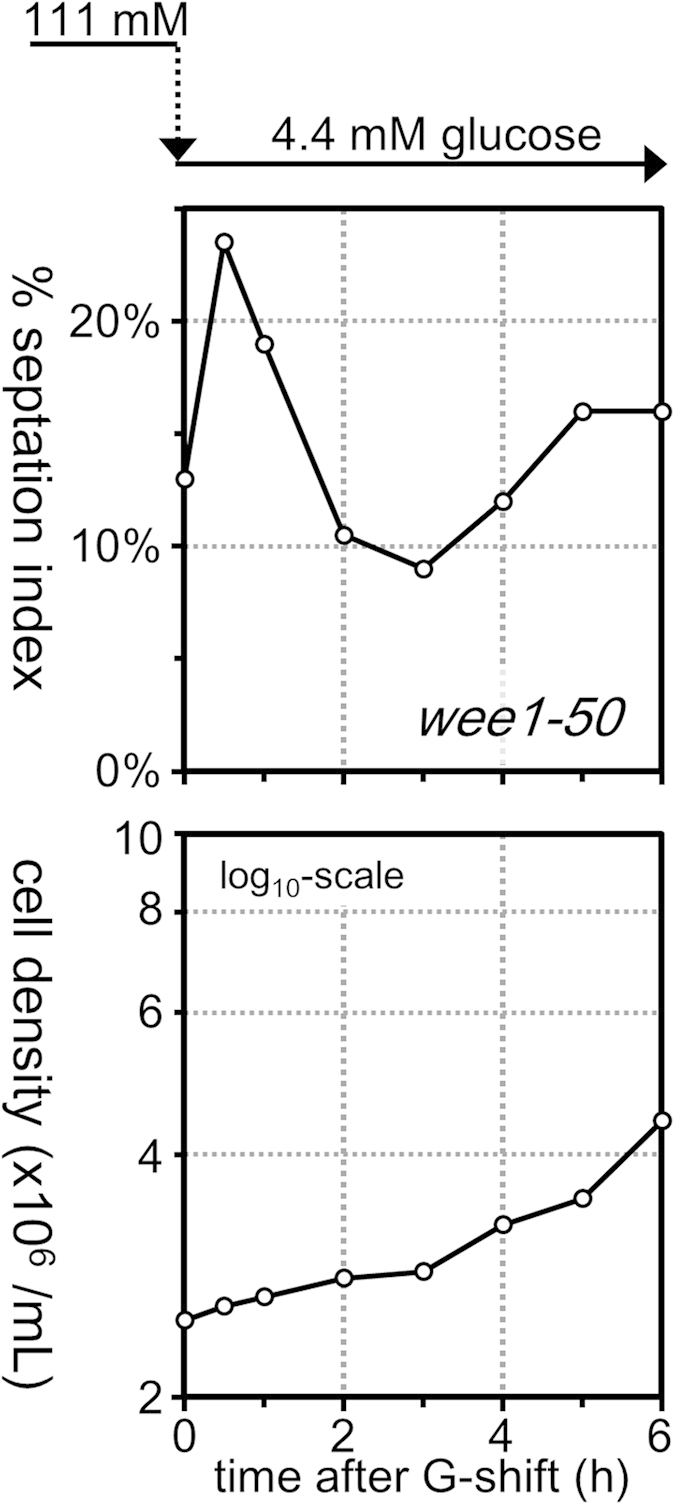
Fully functional Wee1 is required for G2 cell cycle arrest after glucose restriction. Time courses of changes in septation index (upper panel) and cell density (lower panel) of *wee1-50* mutant cells that were transferred from high-glucose (111 mM) to low-glucose (4.4 mM) medium at time = 0 were examined. Cells were cultivated at 26 °C, a permissive temperature for the *wee1-50* mutant, throughout the measurement. For %SI, 200 cells were examined.

To confirm the observations above, %SI was measured in *wee1-50* cells, which harbour a temperature-sensitive mutation in the *wee1*^+^ gene. At a restrictive temperature, 36 °C, the *wee1-50* mutant cells divide at a reduced cell size due to inactivation of Wee1 leading to premature entry into M phase^45^. Transient reduction of %SI was not observed in the *wee1-50* mutant after transfer to low-glucose medium even at a permissive temperature, 26 °C, where the cell length appeared largely normal, suggesting that fully functional Wee1 is required for G2 cell cycle arrest upon glucose restriction (Fig. 3).

### Wee1 is essential for extension of chronological lifespan under conditions of glucose starvation

**Figure 4:**
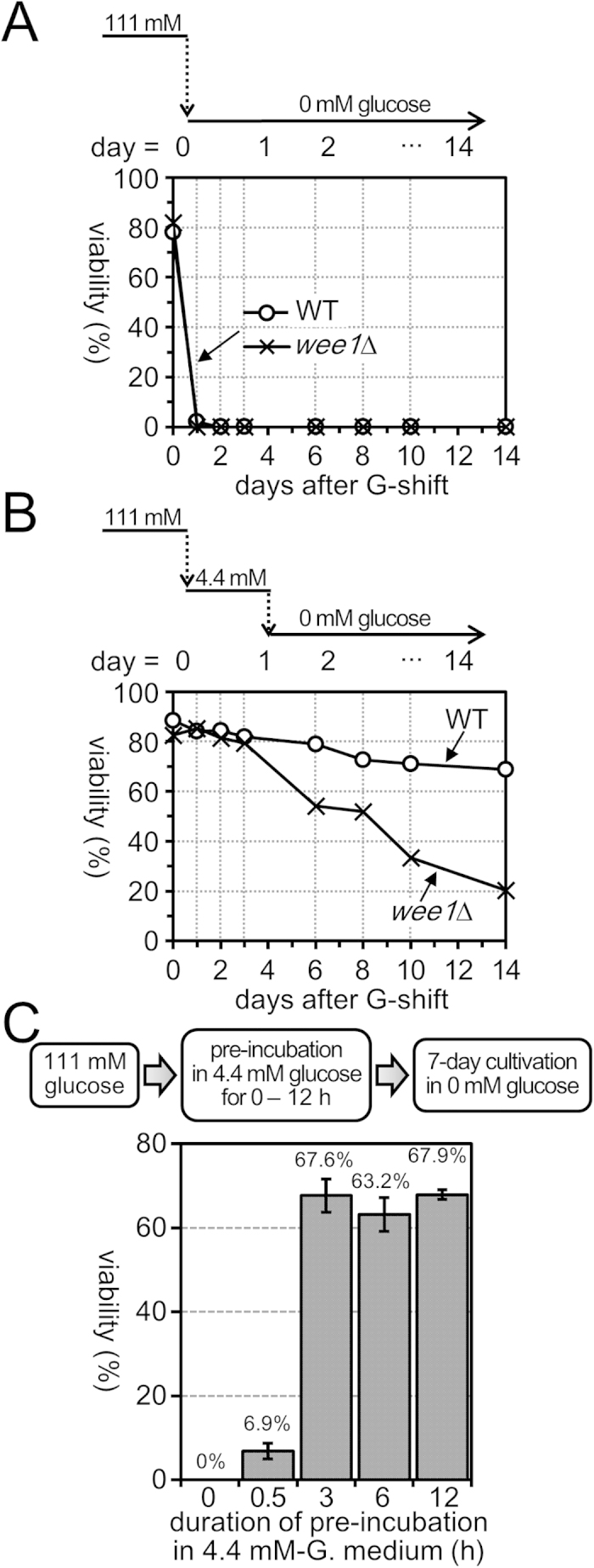
Cultivation in low-glucose medium extends chronological lifespan under glucose starvation in a Wee1-dependent manner. **(A,B)** Time course of changes in viability of cells cultivated in glucose-depleted medium. WT (open circles) or *wee1*Δ (crosses) cells grown in high-glucose (111 mM) medium were transferred to medium lacking glucose directly (**A**) or after 1-day incubation in low-glucose (4.4 mM) medium (**B**). Aliquots of the cell culture were taken at the indicated time points and viability was determined by measuring the proportion of cells forming colonies on solid YES medium. **(C)** Passage of transient G2-arrest period in low glucose is essential for extension of lifespan under conditions of glucose starvation. The experimental procedure is illustrated in the upper panel. The WT cells grown in high-glucose (111 mM) medium were transferred to and cultivated in low-glucose (4.4 mM) medium for 0, 0.5, 3, 6 or 12 hours, and then transferred to medium without glucose. Cell viability was measured after 7-day incubation in glucose-depleted medium. The averages of three independent experiments are shown. The bars indicate ±SD.

The above findings strongly suggest the existence of a novel cell cycle control mechanism involving Wee1, which blocks G2/M transition temporarily in response to abrupt reduction of extracellular glucose concentration from 111 mM to 4.4 mM. This raises questions regarding the physiological consequence of the transient G2 arrest upon glucose restriction. In nature, glucose is thought to be exhausted shortly after its reduction from the environment of the cells, and therefore we suspect that this arrest may be required for cellular adaptation to glucose-limited environments, and preparation for survival in the complete absence of glucose. WT *S. pombe* cells, which were transferred directly from high-glucose (111 mM) medium to medium completely lacking glucose (glucose-depleted medium), lost viability within 3 days^28^ (Fig. 4A), whereas they could maintain high viability for more than 2 weeks in glucose-depleted medium if they were cultivated in low-glucose (4.4 mM) medium for 1 day in prior to transfer^28^ (Fig. 4B). In contrast, *wee1*Δ mutant cells lost viability faster than WT in glucose-depleted medium even after pre-cultivation in low-glucose medium (Fig. 3B); at 14 days after transfer from high-glucose medium, the viabilities of WT and *wee1*Δ cells pre-incubated in low-glucose medium before transfer were 68.8% and 20.4%, respectively. These results indicated that adaptation to low-glucose environments greatly extended the cellular chronological lifespan under conditions of glucose depletion, and that Wee1 plays a pivotal role in this lifespan extension.

We next examined the minimum length of pre-incubation in low-glucose medium required for extension of chronological lifespan under conditions of glucose depletion. WT cells that were pre-incubated in low (4.4 mM)-glucose medium for 0.5, 3, 6 and 12 hours were transferred to glucose-depleted medium, and their viability was measured after 7 days of cultivation without glucose (Fig. 4C). While cells pre-incubated in low-glucose medium for longer than 3 hours retained high viability (~70%), the viability of cells pre-incubated for only 0.5 hour decreased to 6.9%. This result suggested that the full extension of chronological lifespan requires pre-incubation in low-glucose medium for more than 3 hours. Notably, reduction of %SI due to G2 arrest was hardly perceptible at 0.5 hour after glucose restriction, and became most prominent at 2 hours. Therefore, the passage of G2 cell cycle arrest period in low-glucose medium, during which cells supposedly acclimate to glucose-limited environments, may be important for extension of cellular lifespan.

## Discussion

Cell cycle progression is regulated in response to changes in environmental conditions. Our findings strongly suggest the presence of a cell cycle regulatory mechanism that temporarily blocks G2/M transition in response to reduction of extracellular glucose supply. Transient reduction of %SpI and %SI indicates that *S. pombe* WT cells in M phase at the moment of transfer to low-glucose medium complete nuclear division and subsequent cytokinesis before stopping proliferation, whereas the cells in G2 phase do not initiate mitosis. Cells in G1 or S phase appear to finish DNA replication before cell cycle arrest, as the majority of cells contain 2C DNA after glucose restriction. This G2 block due to glucose restriction is thought to be determined genetically, as deletion of the *wee1*^+^ gene, which encodes an evolutionarily conserved tyrosine kinase regulating CDK activities, bypasses this G2 block and causes the accumulation of cells with unreplicated DNA during proliferation under glucose-limited conditions. We propose that this cell cycle regulatory mechanism is a novel type of checkpoint, which monitors glucose availability and allows cells to adapt to glucose-limited environments. During the period of G2 arrest, cells may enhance their capability for glucose transport and mitochondrial ATP generation, and gain energy and carbon source sufficient for completion of mitosis and cytokinesis. This glucose-monitoring checkpoint may, at least in part, extend the cellular chronological lifespan in the absence of glucose, as mutant cells lacking the *wee1*^+^ gene have a shorter lifespan than WT cells. Although further studies are required to determine the mechanism underlying sensing of the amount of glucose uptake, we suspect that the CaMKK (Ssp1 in *S. pombe*) – Protein phosphatase type 6 (PP6, Ppe1 in *S. pombe*) signalling cascade may play a pivotal role in this glucose-monitoring checkpoint. We reported previously that *S. pombe* Ssp1 and the PP6-inhibitor, Sds23, which genetically interacts with Ssp1^46^, are essential for transcriptional elevation of the high-affinity glucose transporter gene, *ght5*^+^, upon glucose limitation^26^. While the Ssp1 protein is localized mainly in the cytoplasm, it moves transiently to the vicinity of the cell surface when cells are exposed to stresses, such as an osmotic stress^47^. The CaMKK signalling pathway may transmit molecular signals from an as yet unidentified glucose sensor on the cell surface to cell cycle regulators, including Wee1, as well as the transcriptional regulator, Scr1^26^.

It is noteworthy that deletion of the *wee1*^+^ gene shortens the chronological lifespan of *S. pombe* cells under glucose-depleted conditions, whereas it does not substantially affect the viability of the cells growing in glucose-rich medium. Although it remains to be determined whether the “glucose-monitoring checkpoint” described above exists in higher eukaryotes, if present, inhibition of Wee1 activity in these organisms may selectively kill cells under conditions of glucose starvation. As microenvironments surrounding tumour cells are likely to contain only a limited supply of nutrients, including glucose, these cells may be selectively killed by treatment capable of inhibiting Wee1 kinase. Therefore, our findings suggest that Wee1 is a good molecular target for cancer therapy.

## Methods

### General techniques and strains

General procedures for handling of *S. pombe* were described previously^26^. For cultivation of *S. pombe* cells, rich yeast extract/glucose/supplements (YES) medium and synthetic minimal EMM2 medium were used with modified glucose concentrations as indicated^48^. Unless otherwise stated, the cells were cultivated at 26 °C. Cell viability was expressed as the ratio of the number of colonies formed on YES solid medium containing 167 mM (3%) glucose to the total number (~1,000) of cell bodies plated. Measurement of cellular DNA content by flow cytometry was performed as described previously^49^. For C-terminal gene tagging and gene disruption, the PCR-mediated method^50^ was employed. The strains used in this study were WT (972; *h*^−^), *wee1*Δ (SP4892; *h*^−^ *wee1*Δ::NAT), WT expressing GFP-labelled α-tubulin and Sid4 (K399; *h*^−^ *lys1*^*+*^::nda2^Prom^-GFP-*atb2 sid4*-GFP::KanMX4) and *wee1*Δ expressing GFP-labelled α-tubulin and Sid4 (K391; *h*^+^ *wee1*Δ::NAT *lys1*^*+*^::nda2^Prom^-GFP-*atb2 sid4*-GFP::KanMX4).

### Fluorescence microscopy

Fluorescence microscopy was performed using an EVOS fl microscope system (ThermoFisher Scientific, Waltham, MA, USA) equipped with 100× (numerical aperture (NA) 1.35) and 20× (NA 0.45) objective lenses, or an Axiovert 200M microscope system (Carl Zeiss, Oberkochen, Germany) equipped with a 100× objective lens (NA 1.40). Calcofluor (10 μg/mL; Sigma-Aldrich, St. Louis, MO, USA) and 4′,6-diamidino-2-phenylindole (DAPI, 50 μg/mL) were applied to cells fixed with 2.5% glutaraldehyde^51^ for fluorescent staining of DNA and septum, respectively. To measure %SI, 400 cells were examined in each sample, unless otherwise stated. To visualize microtubules and SPB, cells expressing GFP-labelled α-tubulin and Sid4 were harvested by vacuum filtration and fixed by immersing in methanol chilled to –80 °C for 30 minutes. The fixed cells were washed with phosphate buffered saline three times before observation. To measure %SpI, 200 cells were examined in each sample.

## Additional Information

**How to cite this article**: Masuda, F. *et al.* Glucose restriction induces transient G2 cell cycle arrest extending cellular chronological lifespan. *Sci. Rep.* **6**, 19629; doi: 10.1038/srep19629 (2016).

## Supplementary Material

Supplementary Information
