# Supplementary material for: Glucose restriction induces transient G2 cell cycle arrest extending cellular chronological lifespan
Source: Sci Rep. 2016 Jan 25;6:19629. doi: 10.1038/srep19629 (PMC4726166; doi:10.1038/srep19629)
Supplement: Supplementary Information [file srep19629-s1.pdf]

Supplementary information

Glucose restriction induces transient G2 cell cycle arrest extending cellular chronological lifespan

Fumie Masuda[1], Mahiro Ishii[2], Ayaka Mori[3], Lisa Uehara[3], Mitsuhiro Yanagida[3], Kojiro Takeda[2,4,*], and Shigeaki Saitoh[1,*]

1) Institute of Life Science, Kurume University, Hyakunen-Khoen 1-1, Kurume, Fukuoka 839-0864, Japan

2) Department of Biology, Faculty of Science and Engineering, Konan University, 8-9-1 Okamoto, Higashinada-ku, Kobe 658-8501, Japan

3) Okinawa Institute of Science and Technology Graduate School, Tancha 1919-1, Onna, Okinawa 904-0495, Japan

4) Institute for Integrative Neurobiology, Konan University, 8-9-1 Okamoto, Higashinada-ku, Kobe 658-8501, Japan

*Authors for correspondence

saitou_shigeaki@kurume-u.ac.jp (Shigeaki Saitoh)

takeda@center.konan-u.ac.jp (Kojiro Takeda)

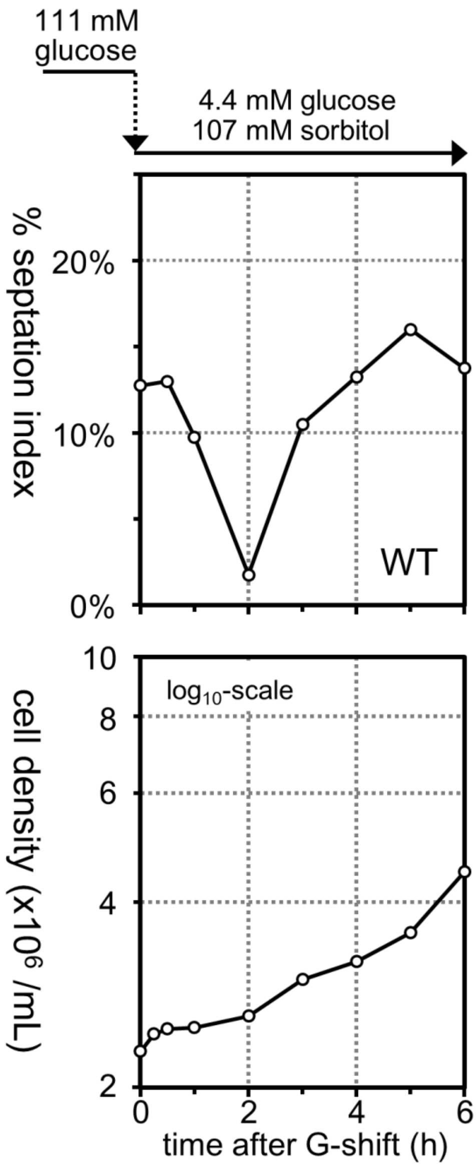

Supplementary Figure 1.

Transient cell cycle arrest after glucose reduction is not caused by hypotonic shock.

Time courses of septation index (upper panel) and cell density (lower panel) of WT fission yeast cells that were transferred from high-glucose (111 mM) EMM2 medium to low-glucose (4.4 mM) medium supplemented with 107 mM sorbitol, which compensated the reduction of osmotic pressure, were examined.
